# Radiographer-led discharge for emergency care patients, requiring projection radiography of minor musculoskeletal injuries: a scoping review

**DOI:** 10.1186/s12873-022-00616-6

**Published:** 2022-04-29

**Authors:** Jenny Shepherd, Ilianna Lourida, Robert M. Meertens

**Affiliations:** 1 Medical Imaging, College of Medicine and Health, University of Exeter, 79 Heavitree Rd, Exeter, EX1 2LU UK; 2 NIHR Applied Research Collaboration (ARC) South West Peninsula (PenARC), University of Exeter Medical School, St Luke’s Campus, University of Exeter, Exeter, EX1 2LU UK

**Keywords:** Radiographer-led discharge, Service improvement, Early discharge, Emergency department

## Abstract

**Background:**

Pressure on emergency departments (EDs) from increased attendance for minor injuries has been recognised in the United Kingdom. Radiographer-led discharge (RLD) has potential for improving efficiency, through radiographers trained to discharge patients or refer them for treatment at the point of image assessment. This review aims to scope all RLD literature and identify research assessing the merits of RLD and requirements to enable implementation.

**Methods:**

We conducted a scoping review of studies relating to RLD of emergency care patients requiring projection radiography of minor musculoskeletal (MSK) injuries. MEDLINE, Embase and CINAHL, relevant radiography journals and grey literature were searched. Articles were reviewed and the full texts of selected studies were screened against eligibility criteria. The data were extracted, collated and a narrative synthesis completed.

**Results:**

Seven studies with varying study designs were included in the review. The small number of studies was possibly due to a generally low research uptake in radiography. The main outcome for four studies was reduced length of stay in ED, with recall and re-attendance to ED a primary outcome in one study and secondary outcome for two other studies. The potential for increased efficiency in the minor MSK pathway patient pathway and capacity for ED staff was recognised. Radiographers identified a concern regarding the risk of litigation and incentive of increased salary when considering RLD. The studies were broadly radiographer focussed, despite RLD spanning ED and Radiology.

**Conclusion:**

There were a low number of RLD active radiographers, likely to be motivated individuals. However, RLD has potential for generalisability with protocol variations evident, all producing similar positive outcomes. Understanding radiography and ED culture could clarify facilitators for RLD to be utilised more sustainably into the future. Cost effectiveness studies, action research within ED, and cluster randomised controlled trial with process evaluation are needed to fully understand the potential for RLD.

The cost effectiveness of RLD may provide financial support for training radiographers and increasing their salary, with potential future benefit of reduction in workload within ED. RLD implementation would require an inter-professional approach achieved by understanding ED staff and patient perspectives and ensuring these views are central to RLD implementation.

## Background

During April 2017 to March 2018 in the UK, there were 23.8 million attendances in the emergency department (ED), a 22% increase since 2008–09. Of these, 12% waited over the target four hours to be discharged or admitted, more than double the expected 5% [1]. In addition, there was a 33% increase of patients attending either minor injuries units (MIU) or walk-in centres during the same time period [1]. To address this increase, it is appropriate to consider radiographer-led discharge (RLD) for patients with minor musculoskeletal injuries [2]. RLD utilises reporting radiographers, trained to either discharge patients with normal images or refer for treatment pathways following pre-specified management plans [3] (Table 1). This innovative pathway was recognised for its potential to reduce the pressure on ED and MIU [2] and first piloted by Snaith in 2007 [3]. Despite being successfully trialled 15 years ago [3], it is still not common practice across the NHS [2].

**Table 1:** Glossary of terms

Radiographer commenting	Radiographer provides written comment on an x-ray, which can be used as a guide, based on their professional opinion [4]
Hot reporting	The radiology report being available at the time the patient leaves the department [3]
Image interpretation	Skill of interpreting x-ray image developed at undergraduate level. Can extend with additional post graduate training to include giving a definitive report on findings [5]
Reporting radiographer	Radiographer trained at Masters level to provide final clinical written reports on x-ray images [3]
Radiographer-led discharge (RLD)	Radiographers already trained to report or interpret images undertaking additional discharge training, either in-house or via emergency nurse practitioner (ENP) course. RLD radiographers give diagnosis and soft tissue injury management information to patients with normal x-rays and discharge them. Patient with abnormal x-rays are referred to the appropriate treatment pathway [2]
RLD criteria	Patients with minor musculoskeletal injuries initially have a clinical examination by a clinician or ENP who refers them for RLD with a likelihood of a management plan for discharge [3]

The NHS plan [6] in 2000 offered the opportunity of role extension for allied health professions (AHPs). Radiographers developed image reporting skills, to the accuracy levels of radiologists [7], leading to improved quality through clinical error reduction [8]. This gave potential for improved efficiency and cost effectiveness; values identified in NHS core principles [6]. Since 2013 image interpretation has been included in undergraduate radiography programmes [9]. This training, extended at post graduate level to advanced practice through Master’s degree programmes, allowed radiographers to report clinically [5]. Nationally in 2017, 78% of hospitals utilised reporting radiographers [5]. RLD also required extension of radiographer training specifically for the discharge process [3].

The Snaith RLD pilot study evidenced a 61% reduction in the patients’ length of stay (LOS) in ED. Patients were discharged or referred for treatment at point of image assessment, by the radiographer with the ED consultant reviewing the outcomes for discharged patients the day after [3]. Hot reporting (Table 1) was also introduced into ED, during the study period, reducing patient recalls, where the radiology report differed with the initial image interpretation, by 52% [3]. The 1.75% patient re-attendance rate also compared favourably to re-attendance rates following discharge by junior doctors (13.1%) and nurse practitioners (8.6%) from other similar studies [3]. However, the pilot also noted there were a further 564 (32%) patients hot reported as normal that RLD was not utilised for, as no management plan was provided at initial assessment [3]. Therefore, with a more robust process there is scope for twofold service improvements with RLD, in line with clinical streaming principles [10]. This is via integrated care benefits and adding value to the patient experience by shortening their journey through ED.

There is potential for improved cost effectiveness in emergency care, with image interpretation errors the leading cause of litigation in ED [2]. For example, use of hot reporting reduced missed fracture litigation claim costs by 66% in one NHS Trust [2]. There could be further cost savings through service streamlining with radiographers discharging patients, increasing ED staff capacity for seeing other patients [2].

A literature review of RLD in 2015 focussed on the impact of RLD on quality of ED services and potential barriers to RLD implementation [11], but did not consider cost effectiveness. Also, the search strategy adopted by the review [11] was not comprehensive. Therefore, it was appropriate to complete a scoping review with additional electronic databases, including grey literature [12]. More recent studies were also available which included ED staff perspectives on RLD, an area for future research identified by the 2015 review [11, 13].

RLD has been recognised as an innovative process [2] and its’ potential demonstrated [3]. Combining the aforementioned studies with recent evidence would allow synthesis of what is currently known about RLD [12]. Thus, conducting a scoping review would be a robust approach, summarising the complete evidence base of RLD for patients with minor musculoskeletal injuries, in emergency care. Synthesising the nature and characteristics of current research would allow identification of further research required to assess the potential feasibility of RLD [12].

## Methods

The PRISMA extension for scoping reviews [14] and framework described by Arksey and O’Malley [12] were used as appropriate tools for this scoping review and formed the basis of the review protocol [14]. Initially, the research question and relevant studies were identified, with the included study selection made using predefined eligibility criteria. Finally, the data from the studies were extracted, charted and summarised [12].

### Identifying the research question

The research question was required to be broader than expected for a systematic review but sufficiently focussed to identify all relevant literature [12, 14]. A primary question was set, based on the Joanna Briggs Institute mnemonic for scoping reviews of population, concept and context [15]. The specific elements included were the population of radiographers and concept of RLD for patients with minor musculoskeletal (MSK) injuries. The context was urgent or emergency care. Key inclusion and exclusion criteria were also developed (Table 2).

**Table 2:** Inclusion and exclusion criteria

Inclusion	Exclusion
**Population**	
Radiographers	AHP professionals other than radiographers
Advance practitioners	Any advanced practitioner who is not a radiographer
**Concept**	
Radiographer-led discharge for projection radiography	Discharge by any other professionals
	Alternative types of discharge i.e. from the body
	Other modalities than projection radiography
**Context**	
Emergency department	GP or outpatient setting. Other healthcare sources which are not acute
Accident and emergency	
Emergency medicine	
**Additional eligibility requirements**	
Articles published post 2000	Articles published pre 2000
Studies within the UK NHS	Private healthcare
Patients with minor injuries	Patients with major trauma
Imaging of appendicular skeleton only	Imaging of the axial skeleton

The international prospective register of systematic reviews (PROSPERO) was searched for a protocol based on this topic, once the question was framed [16]. In addition, Pubmed, Medline and Google scholar were also searched for existing systematic and scoping reviews on RLD. No protocols or existing reviews were found.

### Eligibility criteria

All study designs were considered to add context and depth to the findings, in keeping with a scoping review [12, 14]. As potential barriers for RLD may be linked to implementation and resistance to change [17], rather than feasibility, it was deemed appropriate to include qualitative studies.

The study population was based on the eligibility criteria (Table 2). Therefore, radiographers with advanced training in projection radiography reporting or image interpretation for the appendicular skeleton were included. The concept for inclusion specifically pertained to RLD for minor MSK injuries of the extremities and the context was discharge or treatment referral from emergency or urgent care. As radiographer role extension in the UK was well established following the introduction of the NHS plan in 2000 [6], only UK NHS studies, post 2000, written in English, were included.

### Identifying relevant studies

On the 26^th^ April 2019 CINAHL, Embase and MEDLINE databases were searched. The key words identified in Table 3 and relevant Medical Subject Headings (MESH) terms were combined using Boolean operators. The Radiography journal was hand searched by screening article titles in content pages, for articles pre-dating the March 2017 inclusion on Medline, back to January 2000. The journal was not available online before this date.

**Table 3:** Summary of key words for population, concept and context

radiograph^a^	discharge^a^	emergency department
radiology	patient discharge	accident and emergency
radiographer-led		casualty
		emergency medical services
		emergency service

^a^truncation

Grey literature not available through the traditional databases were also searched [12, 14, 18, 19]. This included sources identified by Public Health England [20], Imaging and Therapy in Practice magazine and the University of Exeter repository (ORE). Keywords used for searching were ‘radiographer-led discharge’ and ‘discharge by radiographer’.

A further search of the included articles reference lists and forward citation chasing was conducted [12, 21]. Scopus medical database and Google Scholar were used for the forward citation chasing [22]. With a limited time scale for the review, a time deadline of the Scopus and Google Scholar searching of 9^th^ June 2019 was set, after which no more new studies were included [12].

### Study selection

Once the search was completed, the citations were uploaded to Endnote software v.X8 (Thomson Reuters, New York, NY, USA) [12] and duplicates removed. A random sample of titles and abstracts were cross referenced by two reviewers [12], to assess inter-assessor reliability. This was a binary check that eligibility criteria had been correctly interpreted, using a percentage agreement check of 10% of the total studies returned from searches.

Once included articles were agreed, full texts were obtained. Initially, three randomly selected articles were independently reviewed by two reviewers, using the full text screening form, based on the inclusion criteria. The reviewers then met to confirm appropriateness of the form [12, 14]. The remaining full text articles were independently screened against the full text form by both reviewers, who were blinded to each other’s results [14].

### Charting and collating the data

The data were extracted using a descriptive-analytical approach and charted under the headings identified by Arksey and O’Malley [12]. The study characteristics were tabulated by aims, design, location, population and intervention, specifically RLD protocol method used. This process allowed emerging themes to be identified [12].  An overview of the characteristics was reported with further synthesis of qualitative and quantitative outcomes included. This thematic charting process identified gaps in the research evidence base, which were reported in the narrative synthesis. Consistent with scoping review methods, study quality was not assessed [12].

## Results

**Fig. 1:**
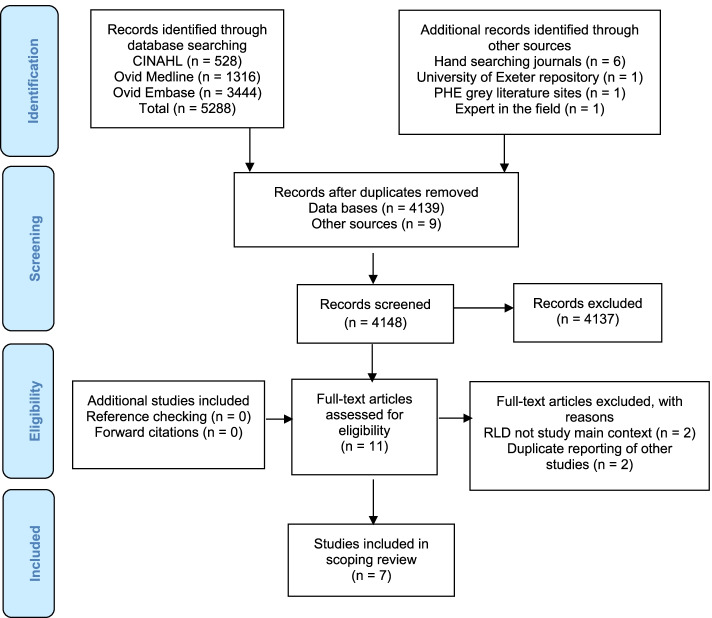
Flow of studies in the scoping review with reasons for exclusion

4148 studies were identified, reducing to 11 following title and abstract screening. Full text screening left nine studies. RLD was not the main context for two articles (Fig. 1). The two reviewers discussed five articles, for potentially duplicate reporting. It was agreed to include three articles, as the reports differed in context.

Three articles were sourced from electronic databases [3, 23, 24]. Hand searching identified one article [4, 11, 25]. One study was located in the ORE [26]. A storyboard was identified through the PHE search [27]. The author was contacted to request related published articles, but no response was received. One article was provided by the author, an expert in the field, following consultation about RLD at the study outset [13].

### Study characteristics

The scoping review process identified a range of study types despite limited study numbers [12, 14]. The studies included an audit [23], two pilot studies [3, 27] a discrete event simulation (DES) modelling study [26], a feasibility study [4], a survey [24] and a mixed methods study [13]. The conference article did not state study type [25] and potentially shared study data with the feasibility study [4]. The modelling and mixed methods articles were possibly based on one study [13, 26]. The mixed methods study was a Health Education England report extending beyond modelling to include interviews, a focus group and training needs analysis (TNA) [13].

There was a geographical spread with two articles based in south west England [13, 26] and two in north east (NE) Scotland [4, 25]. Two studies were based in north east England [23, 24], one in Wales [27] and one in mid-Yorkshire [3]. The review [11] included the NE England [23, 24] and mid-Yorkshire studies [3]. Studies were mostly conducted in general hospital EDs, with the Scottish articles based in community hospital MIUs [4, 25] (Table 4).

Patient numbers in RLD study arms, where stated, ranged from five [27] to 497 [23], with modelling extending to 1303 [26]. The questionnaire uptake was 101 respondents from around 500 contacted [24]. Excluding modelling studies [13, 26], 3 was the maximum number of RLD radiographers per study [3]. Howard identified RLD radiographers driving the process; a relevant concept if radiographer motivation is pertinent to RLD success [4].

Different patient age ranges were included, where stated, with adults included in three studies [3, 4, 23] and paediatrics only in one [27]. Two studies excluded patients under five years old [3, 23] and one study excluded under two year olds [4], whilst Jenkins only included paediatrics [27]. Justifications for paediatric exclusion were difficulty in clinical assessment [3] and potentially more complex symptoms [23].

The primary aim for four studies was reviewing impact of RLD on length of stay (LOS) in the emergency department, either arrival to discharge [3, 25, 26] or x-ray to discharge [23, 27]. The other studies assessed RLD feasibility [4], and radiographer attitudes to RLD [24]. Knapp et al. primarily scoped local reporting radiographer training requirements [13].

Secondary aims varied from impact on ED recall and re-attendance rates [23, 25] to improving patient experience [27] and reviewing use of a DES model as a support tool for using RLD [26] (Table 4).

**Table 4:** Study characteristics

Authors	Primary aim of the study	Secondary aim of the study	Study type and design	Location and site numbers	Study population and participant numbers	Intervention duration, type and comparator
Barter 2015 [11]	RLD impact on quality of ED services and professional practice	Examine barriers and disadvantages of RLD	Review of literature for RLD	Not clearly stated (NCS)	Patients receiving RLD compared with standard discharge, radiographers and radiologists. Participant numbers NCS^a^	All studies of RLD from 2000 until publication, including all interpretations of RLD
Henderson et al. 2012 [23]	Can RLD reduce x-ray to discharge LOS^b^ without impact on patient outcome	NCS^c^ but arrival to discharge LOS was compared in the study. Recall and re-attendance rates	Prospective audit of RLD	1 North east (NE) England General Hospital ED department	> 5 years old. Below elbow/ knee injury, able to weight bear and be discharged after x ray with no follow up. 497 in intervention, 2632 comparators	3 month pilot audit then 2 year audit. RLD defined as reporting radiographer discharging patients, with advice, whose ENP^d^ or Doctor wrote a discharge plan for negative x ray findings at initial consultation. Standard discharge comparator
Howard 2017 [4]	Feasibility of RLD in a community hospital	Explore the impact of RLD in terms of the patient pathway	Feasibility comparing RLD with standard discharge comparator	1 community hospital Minor injury unit in NE Scotland	> 2 years old for extremity musculoskeletal injury below knee & shoulder. Participant number not clearly stated	6 month, RLD process of discharge of patients with minor musculoskeletal injuries, with written radiographer comment of no acute bony/joint abnormality. Radiographer offers advice/ minor treatment. Standard discharge comparator
Howard and Craib 2018 [25]	Assess if RLD reduced patient LOS	Does RLD reduce patient recall or re-attendance rates	Not clearly stated	1 community hospital MIU in NE Scotland	30 patients with no bony injury on x-ray	Duration not clearly stated. RLD process defined as discharge of patients with no bony or joint injury. Standard discharge comparator.
Jenkins 2015 [27]	Can RLD reduce x-ray to discharge LOS, improve patient flow with RLD	Assess if RLD improves overall patient experience	Pilot study of RLD	1 hospital emergency unit in Wales	Intervention 5 children with suspected fractures. Standard discharge comparator of 6 children attending same date and time in previous year	1 afternoon of reporting radiographer using RLD for paediatrics, following competency based 30 h prep including treatment advice and recognising when follow up treatment is required. Standard discharge comparator
Knapp et al. 2016 [13]	Investigate local requirement for reporting radiographers	Review the potential application of RLD	TNA^e^, focus groups, interviews and discrete event simulation for RLD	South west England, 2 site Training needs analysis and 1 site modelling	3 ED interviews. 8 ENPs, 2 ED consultants, 20 radiographers training needs analysis. Focus group with patients/carers. Modelling of ED data matching RLD criteria- number NCS	3 interviews and 1 focus group meeting with researchers. TNA for image interpretation and discharge, numbers NCS. Modelling based on historic data from 2 years. RLD was Not clearly stated
Lumsden & Cosson 2015 [24]	Radiographer attitudes to RLD	Radiographer opinions of salary with RLD	Cross-sectional design survey	7 hospitals across NE England	300–500 questionnaires sent to radiographers. 101 participant uptake	Survey of radiographer views of RLD with no single RLD definition. Timeframe not clearly stated for data gathering
Rachuba et al. 2018 [26]	Use evidence based model to review impact on LOS for RLD suitable patients	Can discrete event simulation modelling be used as a decision support tool for RLD	Discrete event simulation of 2 pathways using RLD	1 South west England district general hospital	Patients who either had minor appendicular injuries or lower limb injuries 1303 in intervention group and 1507 in comparator group	23 months historic data modelling pathways for RLD. RLD defined as; patient with no other condition and normal x ray, discharged with appropriate instructions, and doctor pre-authorisation. Modelling data compared to simulated standard discharge
Snaith 2007 [3]	Assess if RLD could reduce LOS in A&E^f^ pathway	Could hot reporting reduce recall rate	Pilot study of RLD	1 Mid- Yorkshire hospital A&E department	114 patients between 5 and 65 years old, with x ray imaging of distal extremities, excluding knees and shoulders	4 month pilot of RLD, defined as radiographers hot reporting images and discharging patients with advice, using discharge plan written at initial assessment. Standard discharge comparator

^a^not clearly stated, ^b^length of stay, ^c^emergency nurse practitioners, ^d^North East, ^e^training needs analysis, ^f^accident and emergency

### RLD methodology

RLD protocol differed across the studies. The main theme was radiographers discharging patients with normal x-rays, although giving basic treatment advice was also stated in four studies [3, 4, 11, 23, 26]. Three studies required a discharge plan written at initial clinical assessment to be used by the discharging radiographer [3, 4, 23]. Four studies specified extremity only examinations [3, 4, 23, 26], with this detail not stated for the remaining studies.

Two studies modelled RLD pathways with differing variations, based on process mapping within ED [13, 26]. An insightful RLD perspective was also provided by a patient focus group [13]. The survey [24] did not include a standard interpretation for RLD, with the focus being an overview of the RLD concept and radiographer opinion (Table 4).

### Quantitative outcomes

**Fig. 2:**
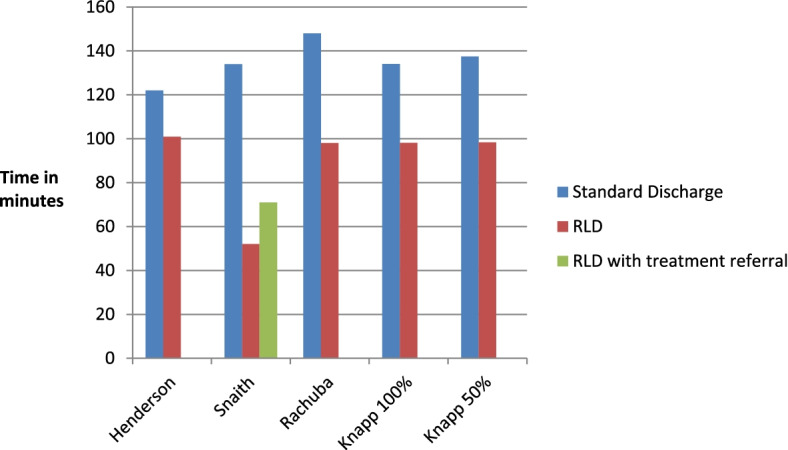
LOS in minutes for RLD compared to standard discharge. Knapp 50% used to align with the other study methods. Knapp 100% modelled continual RLD use [13]

The predominant outcome measure was time of patient arrival in ED to discharge for RLD, compared to standard discharge practice (SDC). Six studies reported a significant LOS reduction using RLD [3, 4, 11, 13, 23, 25–27] although one study omitted numerical data [4, 25] (Table 4). Henderson et al. [23] reported 17% (21 min) mean RLD LOS reduction, compared with SDC, which also included RLD data (Fig. 2). No standalone SDC data was included; however, with no overlap of confidence intervals (CIs) for RLD results were still statistically significant (Table 5). Snaith [3] reported RLD LOS reduction of 61% (82 min) and after RLD treatment referral of 41% (63 min).

**Table 5:** Study results

Authors	Outcome Measures	Results
Barter 2015 [11]	**Primary:** LOS^a^ in ED^b^ with RLD^c^ Vs SDC^d^. Comparing re-attendance rates	Reduced LOS with RLD of 82 min and >20min. Reduced RLD re-attendance 53% and 26.6% for 2 included studies
	**Secondary:** Attitudes to RLD	RLD could reduce LOS and improve services. Concerns over potential for litigation
Henderson et al. 2012 [23]	**Primary:** Overall LOS^a^ in ED^b^ with RLD^c^ Vs SDC^d^	RLD mean 100.9 min. (SD 42.503, 95% CI 97.2 to 104.7). SDC (mean of data during audit, including RLD data) 122 min (SD 48.220, 95% CI 120.3 to 123.7)
	**Secondary:** Clinically significant (CS) false negative results, and re-attendance rates within 28 day period	RLD false negative CS rate 0%. SDC false negative CS rate 1.33%. Odds ratio (OR) false negative ED: RLD 10.59 (95% CI 1.46 to 76.68). RLD re-attendance rate 2.62%, SDC 7.06% with 1.75% CS. OR re-attending with same injury ED: RLD 8.36 (95% CI 2.05 to 34.08)
Howard 2017 [4]	**Primary:** NCS. Patient re-attendance	No patient re-attended
	**Secondary:** Arrival to discharge time	LOS rates were reduced; no numerical data included
Howard and Craib 2018 [25]	**Primary:** Time from arrival to discharge with RLD	RLD reduced length of stay. Minimum journey time 26 min
	**Secondary:** re-attendance or recalls	No re-attendance, one recalled, no management change
Jenkins 2015 [27]	**Primary:** LOS x-ray to discharge with RLD Vs SDC	RLD mean 12.4 min, 72% LOS reduction. RLD with treatment pathway 18 min, 59% LOS reduction. SDC (mean of data from previous year) 44 min
	**Secondary:** Satisfaction surveys	100% satisfaction rating from both staff and patients
Knapp et al. 2016 [13]	**Primary:** TNA^e^ for image interpretation and discharge	Radiographer sensitivity mean 66%, specificity 78%, accuracy 71%. ENP^f^ sensitivity 67%, specificity 54%, accuracy 62%
	**Secondary:** DES^g^ modelling impact on LOS with RLD. Interviews and focus group for RLD	RLD 98.11 min 27% LOS reduction, SDC 134.07 min LOS, using 100% RLD. Interviews—more training required for RLD. Focus group – patient support for RLD
Lumsden & Cosson 2015 [24]	**Primary:** Survey around concept of RLD and comparison of qualitative and quantitative responses	> 70% RLD would help: waiting time targets, LOS in hospital, inter-professional working. 85% stated salary as incentive for RLD. Litigation highest concern (68%)
Rachuba et al. 2018 [26]	**Primary:** modelled **LOS** in ED with RLD with SDC	RLD mean 98 min 66% LOS reduction. SDC 148 min when imaging requested at assessment
	**Secondary:** Analysis of DES modelling pathways for RLD (1) when imaging requested (2) increasing RLD use (3) on different days of the week	(1) Reduction of > 50 min, imaging requested at triage rather than clinical assessment. (2) LOS decreases as RLD increases. (3) Using RLD at weekends, when 51% of all RLD eligible patients present decreases overall ED LOS by average 10%
Snaith 2006 [3]	**Primary:** Overall LOS with RLD Vs SDC	SDC (included patients not requiring imaging) 134 min. RLD no treatment 52 min, 61% LOS reduction. RLD with treatment average 71 min, 47% LOS reduction
	**Secondary:** Number of patients using RLD Vs SDC and patient recall rates	114/1760 (15.9%) used RLD. Recall rate reduced by 52% when compared with data from the same time period in previous years

^a^Length of stay ^b^emergency department ^c^radiographer-led discharge ^d^standard discharge comparator ^e^training needs analysis ^f^emergency nurse practitioner ^g^Discrete event simulation

Note: Standard deviations (SD) and confidence intervals (CI) not reported unless stated

Jenkins [27] piloted x-ray to discharge LOS, with 72% reduction. Henderson [23] also reviewed x-ray to discharge times for RLD, with 12.9 min (SD 9.81) mean LOS. No SDC data was cited (Table 5).

Varying levels of RLD were modelled, compared to simulated standard discharge (SSD). The results reported here used 50% and 100% RLD, compared to SSD. This was formerly to align with the intervention, comparator results from other studies and latterly a more realistic estimate of RLD uptake (Fig. 2). Both results demonstrated reduced LOS, against SSD [13]. Differing days of the week utilising RLD were also modelled. With 51% of RLD eligible patients presenting at the weekend, impact of RLD on these 48 h gave a reduction in ED overall LOS of 10%. The limiting factor for this was only 55% of all ED patients were imaged [26]. Impact on ED capacity was modelled with around 500 RLD discharges allowing time for roughly 300 additional clinical examinations by ED staff [26].

Secondary outcome measures for recall and re-attendance within 28 days from original attendance showed significant reductions. Henderson et al. [23] identified RLD re-attendance rate was 2.62%, and either not clinically significant or unrelated. SDC re-attendance rates were 7.06% of which 1.75% were clinically significant. The odds ratio of re-attending with the same injury through standard discharge, compared to RLD was significant at 8.36 (95% CI 2.05 to 34.08) [23]. However, given the wide CIs, the study may be underpowered and therefore this may introduce uncertainty into the results [28]. Snaith reported RLD re-attendance rates of 1.75% [3], compared against other study results of 13.1% and 8.6% for junior doctors and nurse practitioners respectively [3]. Howard and Craib saw no patients re-attending and one patient recall, with no change in management with RLD [25] (Table 5). No comparisons were drawn against ED senior clinicians, despite them routinely clinically assessing patients and interpreting images. Their level of expertise was likely to lead to fewer imaging requests so was a relevant comparator for future consideration [13].

### Qualitative outcomes

A range of qualitative methods were used [11 13, 24, 27]. The radiographer questionnaire used snowball sampling across hospitals with an estimated 30% response rate [24]. The radiographer respondents were generally positive about RLD, recognising potential benefits. The requirement for salary to reflect the additional training and responsibility was a motivational point [24].

The patient satisfaction survey was 100% positive about RLD [27]. Patients appreciated not having time wasted and A&E staff thought RLD was a good idea and the trial worked well [27]. Knapp et al. used a patient and public involvement (PPI) focus group and ED staff interviews [13]. The PPI group identified positives of reducing waiting times and potential cost effectiveness. The focus group included the benefit of increased patient satisfaction which was also reflected in the patient satisfaction survey responses [27]. The survey was 100% positive about RLD with patients appreciating not having time wasted. ED staff thought RLD was a good idea and the trial worked well [27].

PPI focus group [13] concerns were possible missed diagnosis and increased risk of litigation, the latter also being the main concern in the Lumsden study [24]. Radiographer competency to discharge, patient safeguarding and pathway changes were raised during ED interviews [13]. Appropriate training, competency use and expertise in discharge were discussed in other studies [23, 24].

## Discussion

In this scoping review, seven primary studies were identified addressing RLD in either ED or MIU. This limited number of studies may in part be due to a low uptake of research activity in radiography in general [29].

Differing RLD methodologies were described and those investigating LOS evidenced reductions with RLD, compared to SDC [3, 4, 11, 13, 23, 25–27]. RLD demonstrated potential to both increase clinical assessment capacity for ED staff [3, 13, 27], and efficiency within the minor MSK injuries patient pathway [11, 23, 26]. This was also observed for remote access general practitioners [25]. RLD was a variable protocol-driven process offering potential of generalisability and widespread implementation [30].

Another theme was reduction in image interpretation errors improving recall and re-attendance rates [3, 23, 25]. This could improve patient outcome [31] and decrease likelihood of litigation [32]; a key concern of radiographers surveyed [24]. Radiographer hot reporting has demonstrated cost effectiveness with significant reductions in interpretive errors, compared to ED clinicians [33]. Therefore, RLD cost effectiveness was also likely, combining hot reporting with improved minor MSK injury pathway efficiency.

The studies identified positive outcomes, albeit mostly with short time frames [3, 27] and small sample sizes which could impact the strength of the results [28]. It was important to understand why RLD was not more widely utilised. The concern of litigation has already been identified [24]. A further consideration was radiography culture, where a less supportive work environment could impede role development [34]. At non-RLD sites, radiographers surveyed preferred commenting on images to RLD; this was the reverse for RLD active sites [24]. This could be further explained through resistance by radiographers to extend their practice [34], or less confidence with an unfamiliar process [24].

The small number of RLD active radiographers, up to three [3] per study was noted. RLD radiographers could be considered champions actively promoting the initiative [30], within a supportive culture [34]. They would have resistance to departmental culture issues through belief in RLD [30]. Generalisability of RLD [35] may therefore be reliant on the presence of champions, rather than a concept accepted by all appropriately qualified radiographers [30].

With low RLD radiographer numbers, inconsistent uptake of RLD could be expected [3, 23]. Integration of RLD would require consistent use of the protocol-driven process [30] requiring more RLD radiographers. This was implemented following one study which extended RLD service to evenings and weekends [23]. Pathways of RLD use on different days of the week were also modelled [26]. With 51% of RLD eligible patients attending ED at the weekend; efficient and potentially cost-effective use of RLD could occur on these days [26].

Further themes emerged around inter-professional working [4, 13, 25] and radiographer training in discharge [3, 13, 23, 24]. Radiographers consistently interpreted images more accurately than they expected to [9]. Therefore, future training emphasis requires focus on discharge [3]. Given the radiographers’ concern over litigation [24], use of protocol-driven pathways and appropriate governance systems [2] could encourage engagement.

ED staff could be motivated to support this competency-based training in discharge, once their increased workload capacity was recognised [3, 13, 27]. This capacity was through a decrease in the number of clinical assessments required with increased use of RLD [26]. In addition, ED clinician engagement in protocol development and implementation should reduce the potential of RLD appropriate patients presenting without a management plan [3, 30].

### Strengths and limitations

This is the first scoping review on RLD utilising a comprehensive searching strategy. As such, there is inclusion of both quantitative outcomes and qualitative content allowing contextualisation of the current RLD evidence base.

Ideally there would have been two reviewers at abstract screening stage and reviewing data extraction stages [14]. However, the 10% title and abstract check and full text screening produced full agreement between assessors.

The quality of studies was not assessed [12, 14]. Small sample sizes were identified as limitations [3, 27], with one study having five participants [27]. Larger sample sizes would have increased the power of the study and therefore likelihood of demonstrating true effect of RLD [28]. Henderson [23] included SD and CI in results, which acknowledged variance of LOS, with patients not discharged within the expected four hours [1]. This was omitted by other studies therefore variance of waiting times could impact study results [28].

Potentially there was a further bias with the focus from the radiographer perspective, despite RLD overlapping with ED [25]. Knapp et al. did include interviews with ED staff and PPI focus group, although extending this to ED based studies would address this [13].

### Future research

The narrative synthesis evidenced areas where further investigation could be considered. Reduced recall and re-attendance [3, 23] and service streamlining were identified; however financial impact was not explored. Quality-adjusted life-year (QALY) benefits for patients are possible with increased likelihood of receiving the correct treatment at initial presentation [31]. Hot reporting identified £23.40 saving per patient [5], therefore a good rational for extending DES modelling [26] to a cost effectiveness study of RLD across radiology and ED. Savings could offset some of the training costs and salary increase for radiographers, a motivation for engagement with RLD [24]. A previous study identified radiographer reporting as more cost effective than radiologists. However, further work was required for implementation of the pathway [36]. Innovative thinking between radiology and ED would be required to action the cost effectiveness outcome.

The majority of studies focussed on the radiographer role and Henderson et al. [23] recommended a randomised controlled trial (RCT) as further research. Given the variations in RLD, a cluster RCT with process evaluation would be appropriate to aid fidelity of implementation and give context to outcome variations [37, 38]. This process would include other stakeholders’ perspectives, such as ED staff and patients [6]. As small sample sizes have been identified as study limitations, this would ensure use of larger sample sizes and therefore should give more power to the study [37, 38]. Alternatively, action research – problem solving and improving practice whilst actively undertaking the discharge role, would be an alternative research method, encompassing all relevant parties [38, 39].

Emphasis specifically on the discharge element was required, as the innovative element of RLD [2]. This could be achieved through DES modelling [26] of RLD, from the ED perspective, given the capacity for additional clinical assessments already demonstrated [3, 13, 27]. Alternatively, a time and motion study – monitoring and timing the specific RLD activities, would identify inefficient areas or give improvement targets [40] within the discharge process. This research could directly address the requirement to manage the increasing number of ED and MIU patients [3, 10, 26, 25].

## Conclusion

A limited evidence base of seven RLD studies of varying sample sizes and heterogeneity identified potential benefits for the patient, radiographer and ED. The minor MSK injuries pathway could be streamlined. Journey time through ED and likelihood of recall or re-attendance could be reduced and ED staff may gain increased clinical assessment capacity. A cost effectiveness study could identify RLD financial savings which could contribute towards radiographer training and salary increase; an incentive for engaging with RLD. Further qualitative work to examine the impact of this interdepartmental initiative may be the final key to implementation. Understanding radiography department culture and considering the perspectives of all involved through process evaluations alongside larger quantitative studies could be sufficient to review RLD feasibility. RLD success is not only reliant on radiographer uptake, but dependant on acceptance by both ED staff and patients themselves.

## Data Availability

All data used and analysed during this study are included in this published article.

## Abbreviations

ED: Emergency department
MIU: Minor injuries unit
ENP: Emergency nurse practitioner
LOS: Length of stay
UK: United Kingdom
PHE: Public health England
NE: North East
SDC: Standard discharge comparator
SSD: Simulated standard discharge
SD: Standard deviation
RCT: Randomised controlled trial
RLD: Radiographer-led discharge
NHS: National health service
AHP: Allied healthcare professional
MSK: Musculoskeletal
ORE: Open Research Exeter
TNA: Training needs analysis
DES: Discrete event simulation
CI: Confidence interval
PPI: Patient public involvement
QALY: Quality adjusted life year
