# Correction: Radiographer-led discharge for emergency care patients, requiring projection radiography of minor musculoskeletal injuries: a scoping review

**DOI:** 10.1186/s12873-022-00731-4

**Published:** 2022-10-31

**Authors:** Jenny Shepherd, Robert M. Meertens, Ilianna Lourida

**Affiliations:** 1 Medical Imaging, College of Medicine and Health, University of Exeter, 79 Heavitree Rd, EX1 2LU Exeter, UK; 2 St Luke’s Campus, NIHR Applied Research Collaboration (ARC) South West Peninsula (PenARC), University of Exeter Medical School, University of Exeter, EX1 2LU Exeter, UK

**BMC Emergency Medicine (2022) 22:70**

10.1186/s12873-022-00616-6

The original article [1] contained errors affecting correct attribution of the citations to their respective references which has since been corrected.

